# Clinical and physiological characteristics of tremor in a large cohort of focal and segmental dystonia

**DOI:** 10.3389/dyst.2024.12551

**Published:** 2024-10-09

**Authors:** Zakia Jabarkheel, Aparna Wagle Shukla

**Affiliations:** Department of Neurology, University of Florida College of Medicine, Gainesville, FL, United States

**Keywords:** dystonic tremor, physiology, head tremor, arm tremor, focal dystonia, cervical dystonia, segmental dystonia

## Abstract

**Objective:**

Tremor is a frequent co-occurring feature in patients with dystonia, especially in focal and segmental dystonia. Clinical studies have shown that tremor is more commonly observed when dystonia spreads to contiguous body regions. However, there is insufficient characterization of tremor physiology in focal and segmental forms of dystonia. We aimed to ascertain the characteristics of tremor presenting in these specific subtypes.

**Methods:**

We enrolled dystonia patients with head and arm tremors presenting to our center. We categorized these participants as focal and segmental dystonia following the Movement Disorders Society guidelines. We recorded the frequency, amplitude, rhythmicity, burst duration, and discharge pattern on accelerometer and electromyography recordings. We compared the physiology of tremors in focal vs. segmental dystonia. We determined whether the physiology was affected by clinical features such as demographics, age at onset, dystonia duration, alcohol responsiveness, family history, and botulinum toxin responsiveness.

**Results:**

72 patients, mainly focal cervical dystonia and focal cervical + arm or cranial dystonia (segmental) were enrolled. In the analysis of the head tremor recordings (n = 66; frequency range 3–6.5 Hz), we found that focal vs. segmental dystonia comparisons revealed a significantly lower frequency (mean ± standard deviation; 4.0 ± 0.9 Hz vs. 4.7 ± 1.0 Hz; *p* = 0.02), lower amplitude (0.004 ± 0.008 g^2^/Hz vs. 0.006 ± 0.008 g^2^/Hz; *p* = 0.03) and longer muscle burst durations (111.1 ± 40.4 ms vs. 91.5 ± 24 ms; *p* = 0.04). In the analysis of arm tremor recordings (n = 31; frequency range 3.5–7 Hz), we found focal vs. segmental dystonia comparison revealed a lower amplitude (0.04 ± 0.07 g^2^/Hz vs. 0.06 ± 0.06 g^2^/Hz; *p* = 0.045). In the stepwise regression analysis, the age at evaluation (β - 0.44; *p* = 0.006) and age at onset (β - 0.61; *p* = 0.005) significantly predicted the head tremor frequency whereas the alcohol responsiveness tended to predict the amplitude of the head tremor (β - 0.5; *p* = 0.04) and the arm tremor (β - 0.6; *p* = 0.02).

**Conclusion:**

Our study found that the physiological characteristics of tremor in focal and segmental dystonia are somewhat distinct, suggesting that the spread of dystonia symptoms from one body region to another may have a bearing on the physiology of co-occurring tremor. The frequency of head tremors in younger participants was observed to be higher compared to older participants. The head and arm tremor tended be less severe in patients reporting alcohol responsiveness.

## Introduction

According to many recent clinical studies, tremor is frequently observed to affect patients with dystonia [1–4]. Tremor manifests more commonly in females and is mostly observed during posture maintenance and kinetic tasks, but in some can present even when the body part is at rest [5, 6]. The Movement Disorders Society (MDS) provides guidelines to classify dystonia [7] and tremor according to the clinical and etiological characteristics (clinical and etiological axis). An important classification feature for dystonia is body distribution and the tremor are more common in focal and segmental dystonia compared to generalized or multifocal dystonia. Tremor is even more prevalent when there is a spread of dystonia symptoms [3]. While the optimal definition (or term) for tremor in dystonia requires further refinement, the MDS consensus statement from 2018 describes dystonic tremor as tremor and dystonia affecting the same body part, and tremor associated with dystonia as the tremor and dystonia affecting different body parts [8]. Although a number of studies have reported data on the prevalence of tremors in dystonia, a detailed clinical phenomenological and physiological characterization is lacking. In this study we describe the clinical and physiological characteristics of head and arm tremor observed in a large cohort of dystonia. These patients were categorized into focal and segmental dystonia groups and physiological characteristics were compared. We then examined whether the tremor physiology was influenced by clinical features such as demographics, age at onset for dystonia, duration of dystonia, family history, alcohol responsiveness, and botulinum toxin responsiveness for clinical symptoms.

## Methods

We used an IRB approved protocol to prospectively enroll dystonia patients with a co-occurring tremor presenting at the University of Florida. The diagnosis of dystonia was confirmed by a movement disorder neurologist following the MDS criteria. We assessed the characteristics of head and arm tremor in patients categorized as focal dystonia (symptoms in a single body region) and segmental dystonia (symptoms in two contiguous body regions). The participants were diagnosed with focal cervical dystonia, focal arm dystonia and segmental dystonia comprising of cervical + arm or cervical + cranial dystonia (involving face, jaw, eyes). Head and arm tremors were noted to involve the same or different body regions affected by dystonia. Tremor and dystonia were considered to involve the same body region when there was evident abnormal neck posturing, restricted range of movements and a null point was observed during physical examination in the case of head tremor and features such as arm posturing (splaying and spooning of fingers, thumb hyperextension), shoulder elevation, tremor with a directional character, and a null point was observed in the case of an arm tremor [9–11]. When tremor presented in the contiguous body segment but without the above mentioned dystonic features, we considered the patients to be in the focal dystonia category [12, 13]. For example, focal cervical dystonia patients with arm tremor that was non-dystonic or focal arm dystonia with head tremor that was non-dystonic were categorized as focal dystonia.

Participants were required to withhold their oral medications (at least 8 h) prescribed for treating dystonia and/or tremor and those recruited from botulinum toxin clinic were examined at three or more months after the last round of botulinum toxin injections. We ensured participants did not have comorbidities such as hyperthyroidism, diabetes, and active psychiatric diseases that contribute to enhanced physiological tremor. The clinical characteristics of tremor were recorded during rest, maintenance of posture, and kinetic tasks. Rest tremor was assessed while the participants were lying supine with the head and arms resting. The kinetic head component was assessed when participants were instructed to turn their heads to the extreme right or left, and the kinetic arm component was assessed with participants holding a pen and approximating a dot marked on a sheet placed in front of them. For the postural head component participants were sitting on a chair and instructed to look straight ahead in a neutral position, keeping the head off the wall and trunk of the backrest. The postural arm component was assessed with arms, and hands outstretched at 90° from vertical, keeping parallel to the ground with the palms facing down and the fingers spreading slightly apart. Using the Fahn-Tolosa-Marin standardized rating scale (head and arm tremor items) the tremor amplitude was determined to be mild (score 1), moderate (score 2), and severe (score 3 or 4). Further characteristics such as whether the tremor was fine or coarse, or rhythmic or jerky were determined based on clinical visual assessment.

### Electrophysiology setup and data acquisition

We used the Trigno^™^ Wireless system (Delsys, Inc., Massachusetts) consisting of triaxial orthogonal accelerometers for tremor frequency, amplitude, rhythmicity, and sensors for computing electromyography (EMG) burst duration and the discharge pattern. Participants were seated comfortably in an upright chair with a backrest and an armrest. We recorded the physiology of the head and the arm tremor when maintaining a steady posture for 30–60 s (postural component of the tremor). Sensors were mounted over the glabella and on the dorsum of the most affected hand at 1 cm distance proximal to the third metacarpophalangeal joint to capture the accelerometer data. Sensors were also mounted over the agonist and antagonist muscles of the neck (sternocleidomastoid and splenius capitis muscles) and over muscles of the most affected arm (flexor carpi ulnaris, flexor carpi radialis, and extensor carpi ulnaris and extensor carpi radialis) to capture the surface EMG signals. The location for sensor placement was guided by bony landmarks and muscle palpation during active flexion, extension and rotation of cervical joints and flexion and extension of the elbow joints. The placement was further confirmed with inspection of EMG output recorded with Delsys, EMG works software. We ensured there was a consistent sensor placement across individuals. Data for head and arm tremor physiology recorded over three trials was individually analyzed. In a subset of patients with dystonic arm tremor (n = 8), weights (500 g and 1,000 g) were strapped to the dorsum of the hand for examining the effects of inertial loading on the tremor frequency.

### Electrophysiology analysis

The EMG data from the sensor was sampled at 1926 Hz, amplified, digitized, and filtered at 20–450 Hz. The raw accelerometer signal was sampled at 148 Hz, digitized and filtered (0–50 Hz), and analyzed to calculate frequency peak, spectral power for amplitude, and half-peak bandwidth of the frequency peak for quantification of rhythmicity. EMG data recorded during three trials was visually inspected, and data contaminated with noise signals was excluded from the final segment selected for offline analysis. We assigned onset and offset markers manually to the EMG bursts for calculation of muscle burst duration. We averaged the EMG burst duration across all muscles for the head tremor and the arm tremor at the participant level and the group level. We coded the pattern of agonist and antagonist EMG discharges as a co-contraction pattern, alternating pattern, or mixed pattern (neither co-contraction nor alternating).

A commercial software (EMG Works analysis) performed Fast Fourier transform (FFT) analysis with the Welch method to generate the frequency peak (auto spectra), also known as power spectral density (PSD). A select data series (10-second epochs) was first divided into overlapping sections of a specified window length and window overlap. Then the squared magnitude of the FFT computed for each section was averaged and zero-padded to identify the dominant frequency peak. A baseline shift sometimes observed in raw accelerometer signals due to a limb sway relative to the gravity (de trending and re-zeroing) was calculated with a PSD script [14]. A peak spectral power for the tremor was derived off-line by squaring and summating the peaks of frequency power in x, y, and z-axes and calculating the square root of the summated power [15]. A cycle-to-cycle variability in the frequency was achieved by calculating half-peak bandwidth; width of the spectral peak at one-half the peak amplitude in the power spectrum (wider bandwidth of frequency peak indicating a more irregular tremor) [16]. The analysis of tremor physiology was performed by investigators blinded to the clinical findings.

Statistical analysis for the physiological data was performed using SPSS version 28 with significance set to a threshold of *p* < 0.05. The mean, standard deviation (SD), and range for each physiological measure was calculated at the individual and group level. Based on normality distribution assessed with the Shapiro-Wilkes test, we used non-parametric tests such as Mann-Whitney test for the focal vs. segmental dystonia and tremor and dystonia affecting same or different body region comparisons. We used stepwise linear regression analysis with bootstrapping (to account for skewed distribution) to determine the effects of demographics and dystonia characteristics (age at onset and evaluation, dystonia duration, alcohol responsiveness, family history, and botulinum toxin responsiveness) on the tremor physiology (frequency, amplitude, half-peak bandwidth, and EMG burst duration). The type I error rates for multiple comparisons were also corrected with Holm-Bonferroni method, which adjusts *p* values for each hypothesis with a range of significance thresholds (0.01–0.008).

## Results

### Patient characteristics

**FIGURE 1:**
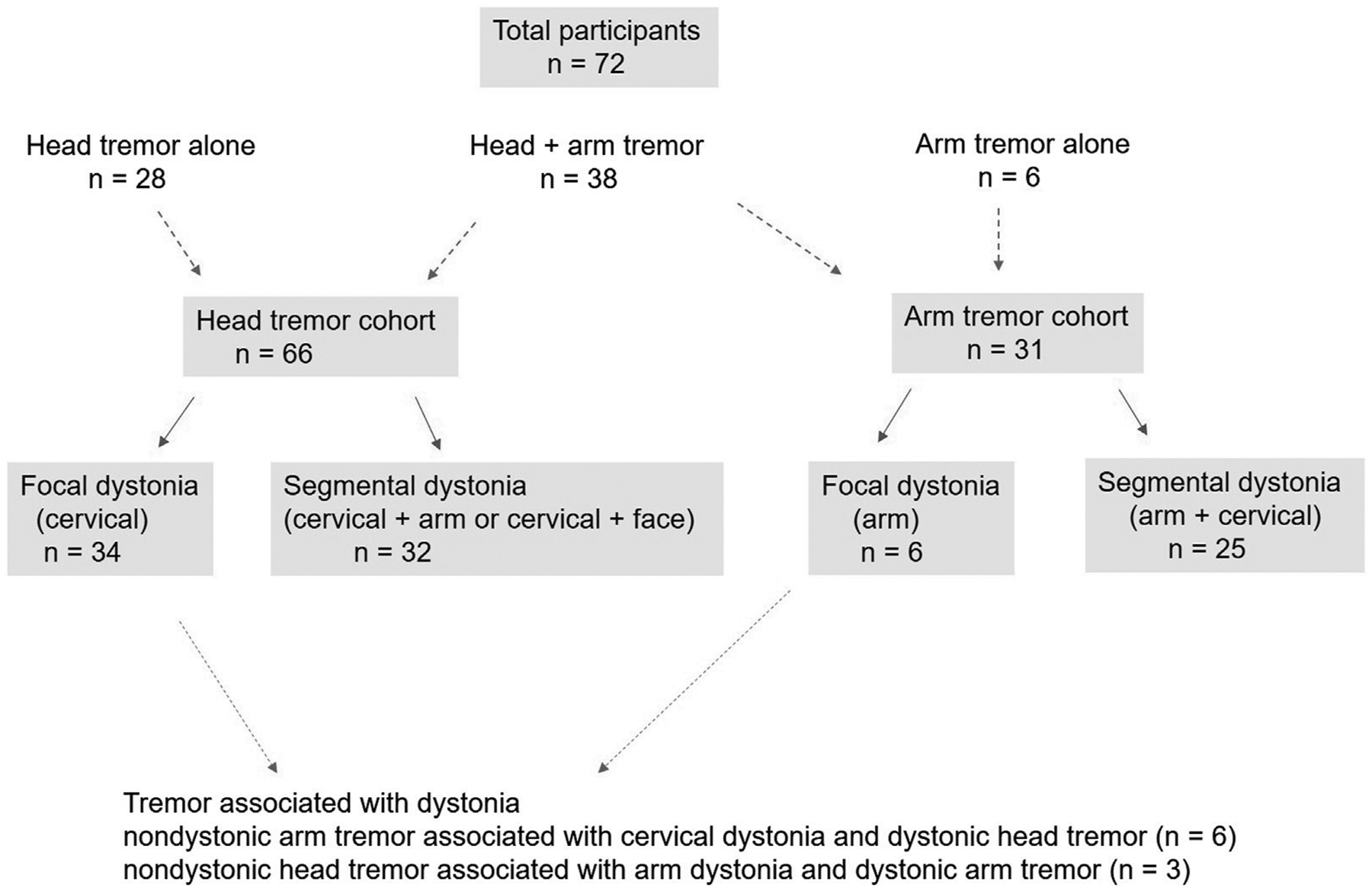
The figure illustrates the participants enrolled in the cohort with head tremors, arm tremors, or both. It shows the distribution of participants into focal and segmental dystonia categories. The figure also shows the number of patients meeting the criterion for tremor associated with dystonia.

72 patients (8 males, 64 females) participated. There were 28 patients with head tremor alone, 38 patients with head + arm tremor and six patients with arm tremor alone (66 patients or 91% with head tremor and 31 patients or 36% with arm tremor). Based on the body distribution of dystonia, these patients were classified into focal and segmental dystonia categories (Figure 1; Table 1). While the majority of patients would be classified as having dystonic tremor, only a few fit the category of tremor associated with dystonia. These patients had nondystonic arm tremor associated with cervical dystonia and dystonic head tremor (n = 6) or nondystonic head tremor associated with arm dystonia and dystonic arm tremor (n = 3).

The mean (±SD) age for the cohort was 67.1 ± 9.2 years, the mean age at onset for dystonia symptoms was 49.5 ± 16.1 years, and the mean duration of symptoms was 17.6 ± 12.4 years. Most participants (n = 68) reported that tremors presented around the same time as dystonia symptoms. Dystonia manifested before tremor for three patients and tremor manifested before dystonia for two patients; however, the time interval between the two clinical features was less than a decade. 60 out of 72 patients were recruited from our botulinum toxin clinic and 90% of patients endorsed improvements with botulinum treatments. Nearly 50% of the cohort reported a positive family history for dystonia, 25% reported their tremor improved with alcohol (subjective self-report) and 75% reported improvement with botulinum toxin injections. The clinical profile for the participants categorized as focal and segmental dystonia (mostly similar) is presented in Table 1.

In the clinical assessment of head tremor, there were 34 patients with focal cervical dystonia (6 patients had nondystonic arm tremor) and 32 patients with segmental dystonia (cervical + arm or cervical + face). A postural component was observed in all 66 patients (80%), kinetic component in 57 (80%) patients, whereas the resting component was seen in 25 (37%) patients. Head tremor was mostly mild (54%) or moderate (28%) in intensity and had a fine and rhythmic character in nearly 2/3rd (68%) of the cohort. Head tremor manifested before arm tremor in more than 75% of patients.

In the clinical assessment of arm tremor, there were six patients with focal arm dystonia (3 patients had non-dystonic head tremor) and 25 patients with segmental dystonia (arm + cervical) Arm tremor was distal in distribution in more than 90% of patients. The postural component was seen in all 100% of participants, the kinetic component in 80%, and the resting component seen in only 36% of the patients. More than 30% of patients presenting with arm tremor had a unilateral tremor. In patients with bilateral tremors, more than 90% of patients had a remarkably asymmetric tremor (amplitude difference between the two arms greater than two points). More patients had a fine, rhythmic or sinusoidal tremor compared to coarse, irregular or jerky arm tremor.

### Physiological characteristics of head and arm tremor

**FIGURE 2:**
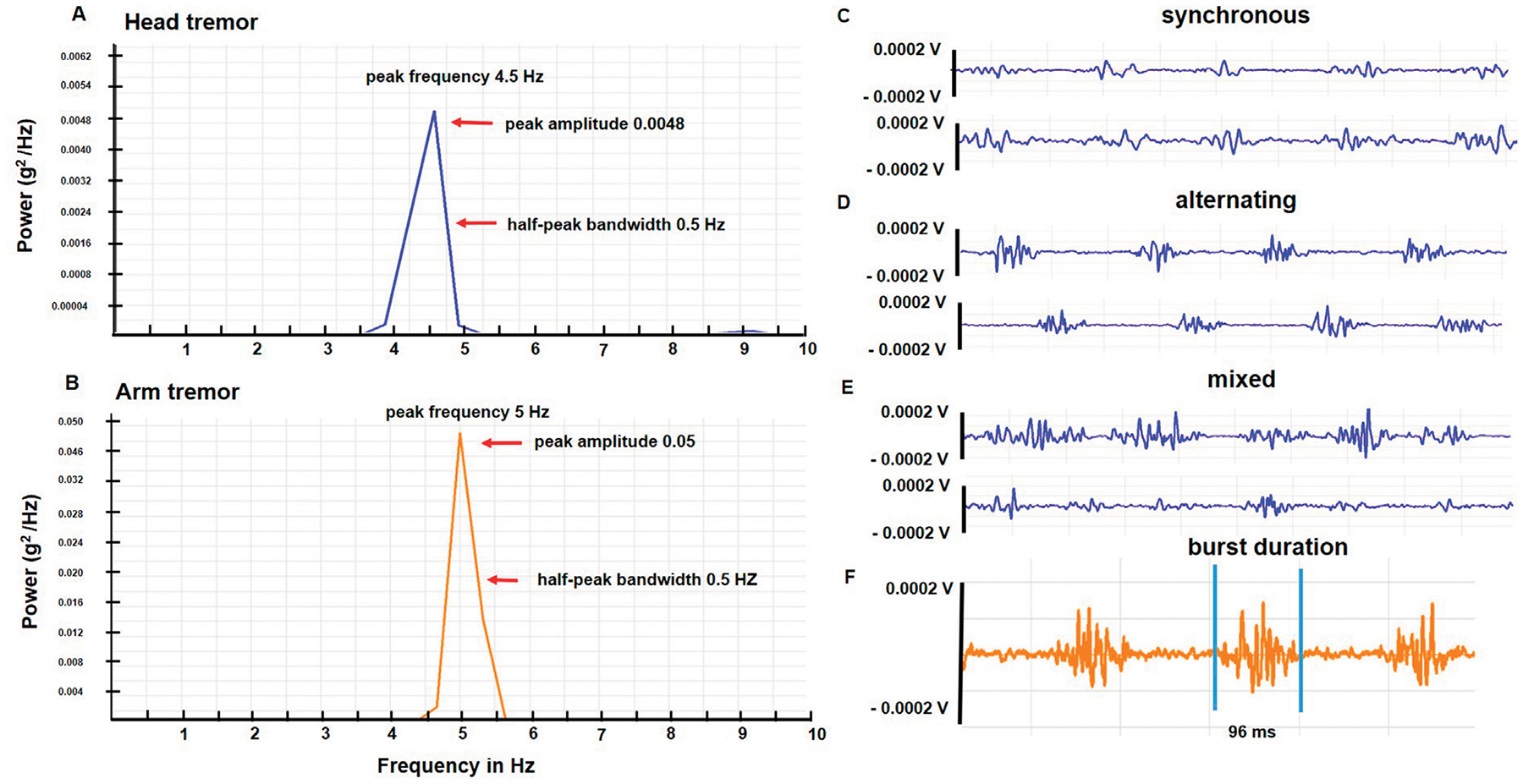
**(A, B)** illustrate power spectrum analysis for the head and arm tremor accelerometer recordings, respectively conducted in one of the participants. The arrows point to the peak frequency, amplitude, and half-peak bandwidth. **(C**–**E)** are sample EMG tracings from a patient with a dystonic head tremor that illustrates the three discharge patterns (synchronous, alternating, and mixed) **(F)** Reveals the burst duration of EMG discharge from a sample arm tracing.

The physiological characteristics of head and arm tremors are charted in Table 2. The mean ± SD frequency for the head tremor was 4.4 Hz ± 1.0 (range 3–6.5 Hz). While the study aims did not involve direct comparisons between head and arm tremor, the accelerometer-based frequency of head tremor (4.4 ± 1.0 Hz; range 3.3–5 Hz) was slightly lower than the arm tremor (5.3 ± 0.9 Hz; range 3.5–7 Hz), the accelerometer amplitude for the head tremor (0.005 ± 0.009 g^2^/Hz) was lower than the arm tremor (0.05 ± 0.7 g^2^/Hz) and the average duration for EMG bursts was shorter for the neck muscles (101.5 ± 31.5 ms) compared to the arm muscles (128.5 ± 39.3 ms). However, the head tremor and the arm tremor had similar half peak bandwidth (0.55 ± 0.09 Hz). In the EMG recordings, three patterns of contractions were observed in the agonist-antagonist pair: synchronous or co-contraction pattern, alternating contraction, and a mixed discharge pattern (a combination of co-contraction and alternating pattern). In more than 80% of the patients, mixed pattern was the dominant pattern for both head and arm tremor recordings. Inertial loading at the wrist did not change the arm tremor frequency but lowered the amplitude measured with the accelerometer and EMG. Figure 2 illustrates the power spectrum analysis of head tremor and arm tremors and EMG tracings recorded from one of the participants.

### Comparisons of focal vs. segmental dystonia

Focal dystonia vs. segmental dystonia comparisons revealed that the head tremor frequency (4.0 ± 0.9 Hz vs. 4.7 ± 1.0 Hz; *p* = 0.01) and amplitude (0.004 ± 0.008 vs. 0.006 ± 0.008; *p* = 0.015) was lower and the EMG burst duration longer (111.1 ± 40.4 ms vs. 91.5 ± 24 ms; *p* = 0.04). Furthermore, comparisons for the arm tremor data revealed a lower amplitude (*p* = 0.045) in focal dystonia (0.04 ± 0.07) compared to segmental dystonia (0.06 ± 0.06). The remaining data comparisons for tremors did not reach statistical significance.

### Comparisons when tremor and dystonia involving same or different body regions

We had six patients with focal cervical dystonia and non-dystonic arm tremor, all of whom also exhibited head tremor. Similarly, we had three patients with focal arm dystonia and non-dystonic head tremor, and these patients also experienced arm tremor. When comparing patients with tremor physiology affecting the same or different body regions impacted by dystonia, our analyses did not produce significant findings, except for a notable finding regarding the head tremor frequency (4.0 ± 0.9 vs. 5.0 ± 0.3; *p* = 0.01) (Table 3). However, considering the highly uneven sample sizes in the two comparison groups, the reliability and validity of the results will need to be interpreted with caution.

### Factors impacting physiology of head and arm tremors

**FIGURE 3:**
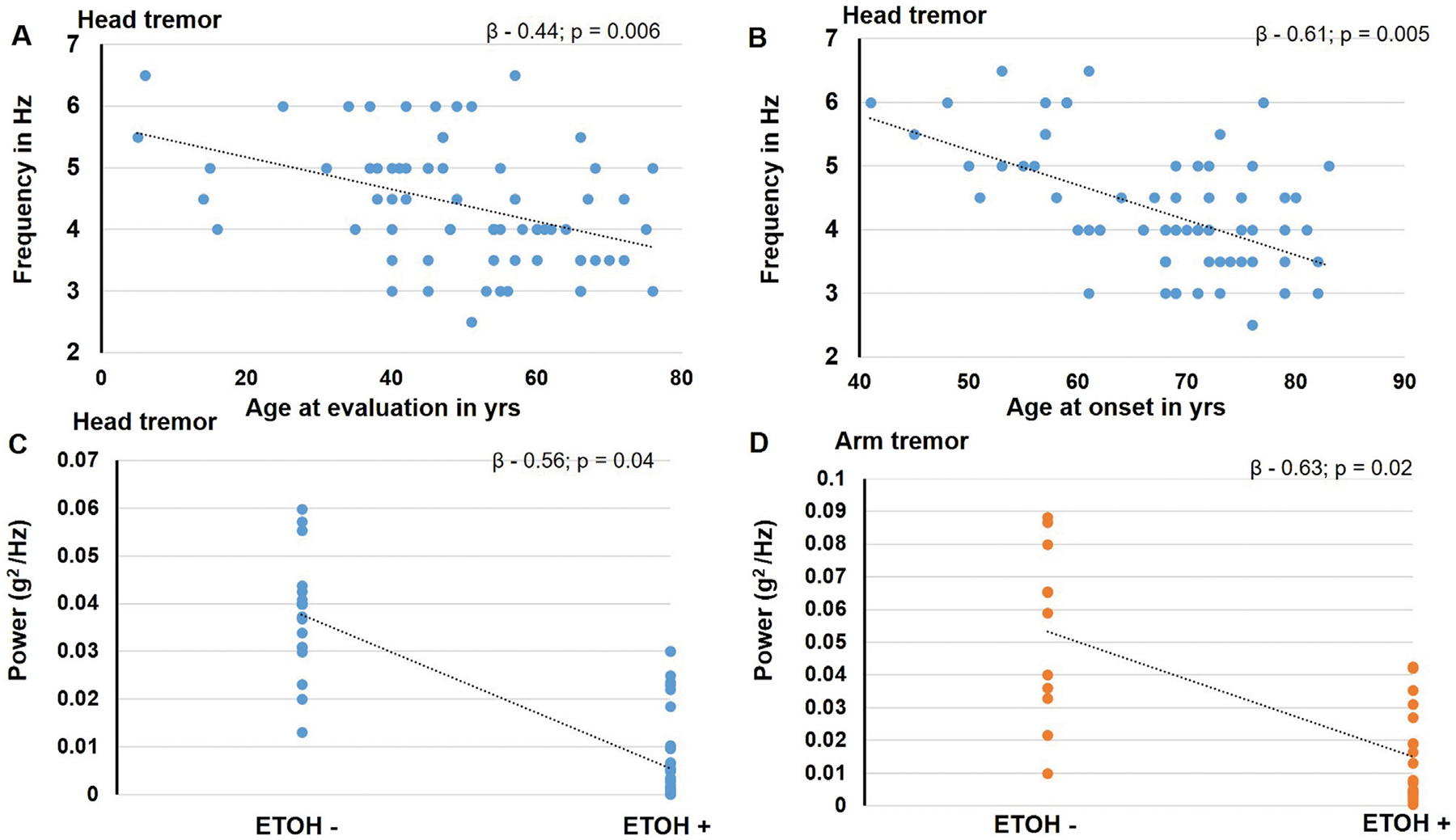
The figure illustrates the findings noted to be significant in the stepwise regression analysis. The blue circles represent data for head tremor findings, and the orange circles represent data for the arm tremor findings. The age at evaluation **(A)** and age at onset **(B)** is found to predict the head tremor frequency significantly. Alcohol responsiveness is found to significantly predict the amplitude of the head tremor **(C)** and the arm tremor **(D)**.

In the stepwise regression analysis, the age at evaluation (β - 0.44; *p* = 0.006) and age at onset (β - 0.61; *p* = 0.005) significantly predicted the head tremor frequency whereas the alcohol responsiveness tended to predict the amplitude of the head tremor (β - 0.5; *p* = 0.04) and the arm tremor (β - 0.6; *p* = 0.02). Figure 3. The other physiological features were not observed to have significant predictors.

## Discussion

Our cohort mainly comprised of cervical dystonia, there was a preponderance of middle-aged females, 25% of patients reported alcohol sensitivity, and most patients reported simultaneous onset around 50 years of age for tremor and dystonia symptoms. These findings are consistent with those reported in the past [5]. While patients consistently presented with a postural and kinetic component, the resting component for tremor was seen for patients presenting with a head and/or an arm tremor in nearly 40% of patients as reported in previous studies [6, 15]. The head tremor manifested before the arm tremor in the majority of patients, and the arm tremor was distinctly unilateral in a third of patients. Patients in our cohort were relatively younger, had tremor onset at an earlier age, and had a longer duration of symptoms than earlier reports [3].

Our data analysis confirms that tremor manifesting in dystonia tends to have a low to medium range frequency (4–5 Hz), which is in keeping with the MDS consensus statement [17]. We also found that tremor arises from medium duration (~100 ms) muscle bursts and a mixed pattern of muscle discharges as the dominant pattern. We also found that the frequency of head tremor was notably higher in younger individuals when compared to their older counterparts. Additionally, individuals reporting alcohol responsiveness tended to experience less severe head and arm tremors.

### Tremor in focal dystonia vs. segmental dystonia

Natural history studies have ascertained that dystonia patients with focal onset symptoms can experience a spread of symptoms into contiguous body regions during the course of their disease [18] The spread of dystonia symptoms occurs in greater than 20% of patients, and the risk of spread is higher in patients who have a tremor [12, 19, 20]. An important goal of the study was to probe whether the clinical nosologic classification into focal and segmental dystonia categories also reflected a distinct physiological segregation, as this would facilitate development of more specific treatments in future [10]. Our study found that many physiological aspects of tremor in focal dystonia such as the frequency, amplitude and muscle burst duration of the head tremor and the amplitude of the arm tremor was distinguishable from segmental dystonia. Future imaging studies are necessary to elucidate the brain networks specific to focal and segmental dystonia. Although the brain networks for dystonia and tremor are likely distinct, they probably interact to some extent, considering they share anatomical structures such as the cerebellum and motor cortex. Thus, alterations in the function of the dystonia network could potentially influence the underlying pathophysiology of tremor. For example, functions of the cerebello-thalamo-cortical pathway might be more involved in segmental dystonia and these may explain our findings of differing tremor physiology in focal dystonia compared to segmental dystonia. Future studies could shed light on the networks that correspond to specific forms of dystonia. In our study, we examined if the physiology was impacted whether tremor and dystonia involved the same or different body parts. Some researchers are concerned that categorizing patients as dystonic tremor or tremor associated with dystonia may not necessarily identify distinct pathophysiological differences [5, 8]. Similarly, in our study, we did not observe significant differences when tremor and dystonia affected the same or different body regions. However, as noted in the results, the uneven distribution of samples limits the strength of these conclusions.

### Factors influencing the tremor physiology

We found an inverse relationship between the age at evaluation and the frequency of head tremor, which is similar to essential tremor literature that found the frequency of the tremor decreases with increasing age [21]. We also found that the presence of alcohol (or ethanol) sensitivity tended to be associated with a lower amplitude of the head and arm tremor. In a recent large study involving over 1,000 patients with dystonia, the presence of alcohol responsiveness was seen in nearly 30% of patients with cervical dystonia and was particularly noted in patients with a co-occurring tremor [22]. While the mechanisms underlying the effects of ethanol in dystonia are not known, these have been studied in essential tremor and have been attributed to increased firing of Purkinje cell neurons of the cerebellum through presynaptic effects and decreased firing of the dentate neurons through postsynaptic effects [23–25]. These potential mechanisms could be extended to the dystonia population as there is evidence to support an underlying dysfunction in cerebellum [26–28]. In our recent functional MRI study, the blood oxygen level-dependent activity in the cerebellum and connectivity between the cerebellum and other brain regions was significantly reduced in patients with dystonia and tremor [29]. Thus, the relationship between alcohol responsiveness and the tremor amplitude seen in our study is likely related to the modulation of dentate nucleus pathway of the cerebellum.

Our study examined the effects of inertial loading to determine whether the tremor had a mechanical-reflex component. Previous inertial loading studies found that the mechanical-reflex component could be separated from the 8–12 Hz central component (synchronous modulation of motor unit discharges that are central in origin) in patients with a physiological tremor. In the power spectral analysis, there was an emergence of the mechanical-reflex peak separate from the 8–12 Hz central peak. However, such a separation of two frequency peaks was not seen in essential tremor and Parkinson’s disease tremor, lending credence to a central origin for these tremors [30–32]. McAuley et al. found a lowering of the arm tremor amplitude with inertial loading which was also seen in our cohort. However they found the separation of mechanical and central frequencies in two of the six patients studied [33], which was not seen in our patients. These discrepancies could be related to differences in the comorbidity burden; we specifically excluded conditions that could lead to a co-occurring enhanced physiological tremor.

Our study has many strengths, given that the data was collected from one of the largest and well-characterized cohort of patients. Our study advances the physiological understanding of tremor manifesting in dystonia, which can potentially lead to more effective treatments. For example, in patients treated with deep brain stimulation of the ventral intermedius nucleus of the thalamus [34], the selection of stimulation frequency could be adapted and optimized based on the frequency of tremors in keeping with closed-loop neuromodulation principles [35, 36]. Then, differences in tremor physiology between individuals could be leveraged in understanding variation in the treatment response to neuromodulation. As new drugs are being investigated for treatment, treatments based on neurophysiological characteristics might emerge instead of clinical characteristics. Indeed, a third pathophysiology-based axis of classification has been proposed to guide the effective management of patients [37].

We acknowledge that our study has several limitations, including the lack of longitudinal recordings and the purely clinical assessment of certain characteristics, such as jerky or rhythmic tremors. Additionally, the study lacks physiological assessment of the resting and kinetic components of tremor and does not include tremors in other body parts, such as the jaw and legs. As recommended by the MDS, a sub-classification based on the age of onset for dystonia or temporal pattern of dystonia was not given due consideration. While the recordings were performed off medications, we have not assessed the response of physiological characteristics to medications. Finally, regarding the analysis, we have yet to determine the coherence between signals recorded from homologous muscles of the two sides, as most recordings were for the head tremor and the arm tremor recordings were unilateral in many patients.

In summary, our research identified a significant prevalence of tremors in both focal and segmental dystonia. These tremors were predominantly postural/kinetic, featuring some rest component, and exhibited a tendency towards fine and rhythmic characteristics rather than coarse and jerky movements. The observed distinctions in tremor physiology between focal and segmental dystonia categories indicate that the distribution and spread of dystonia symptoms play a role in shaping tremor features. Our findings also suggest that an earlier age of symptom onset is linked to a higher frequency of head tremor, and alcohol-responsive head and arm tremors tend to be milder. It is important to note that these hypotheses require further examination in larger cohorts. Nevertheless, the intriguing connection between tremor and dystonia networks, along with the impact of disease progression, warrants further research. Tracking these aspects could be achieved through future longitudinal natural history studies.

## Figures and Tables

**TABLE 1:** Demographics and clinical profile and clinical characteristics of tremor.

Demographics and clinical profile	n = 72
Age in years, mean ± SD	67.1 ± 9.2
Sex, male/female	8/64
Age at onset in years, mean ± SD	49.5 ± 16.1
Disease duration in years, mean ± SD	17.6 ± 12.4
Alcohol responsiveness, n (%)	19 (26)
Family history of dystonia, n (%)	33 (46)
Botulinum toxin responsiveness, n (%)	56 (78)

	Total	Focal dystonia	Segmental dystonia
**Head tremor characteristics**	n = 66	n = 34	n = 32
Rest/Posture/Kinetic (n)	25/66/57	12/34/32	13/32/29
Mild/Moderate/Severe (n)	39/20/7	14/6/4	20/11/1
Fine/Coarse (n)	43/23	21/13	22/10
Rhythmic/Jerky (n)	45/21	25/9	20/12
**Arm tremor characteristics**	n = 31	n = 6	n = 25
Rest/Posture/Kinetic (n)	13/31/25	5/6/6	6/25/20
Mild/Moderate/Severe (n)	5/22/4	2/3/1	8/15/2
Fine/Coarse (n)	17/14	3/3	14/11
Rhythmic/Jerky (n)	14/12	7/3	8/8
Unilateral/Bilateral (n)	11/20	3/3	8/17
Symmetric/Asymmetric (when bilateral) (n)	5/15	1/2	5/12

**TABLE 2:** Electrophysiological characterization of head and arm tremors.

	Participants	Focal dystonia	Segmental dystonia	p-value *indicates significance
**Head tremor**	n = 66	n = 34	n = 32	
Frequency in Hz, mean ± SD	4.4 ± 1.0	4.0 ± 0.9	4.7 ± 1.0	0.01*
Amplitude in g^2^/Hz, mean ± SD	0.005 ± 0.009	0.004 ± 0.008	0.006 ± 0.008	0.015*
Half peak bandwidth/irregularity in Hz, mean ± SD	0.6 ± 0.41	0.5 ± 0.24	0.6 ± 0.42	0.37
EMG burst duration in ms, mean ± SD	101.5 ± 31.5	111.1 ± 40.4	91.5 ± 24.1	0.04*
EMG pattern, synchronous/mixed/alternating	7/50/9	3/25/6	3/23/6	
**Arm tremor**	n = 31	n = 6	n = 25	
Frequency in Hz, mean ± SD	5.3 ± 0.9	5.2 ± 0.9	5.3 ± 1.1	0.35
Amplitude in g^2^/Hz, mean ± SD	0.05 ± 0.7	0.04 ± 0.07	0.06 ± 0.06	0.045*
Half peak bandwidth/irregularity in Hz, mean ± SD	0.5 ± 0.49	0.5 ± 0.44	0.5 ± 0.32	0.61
EMG burst duration in ms, mean ± SD	128.5 ± 39.3	127.2 ± 40.2	130.3 ± 38.1	0.21
EMG pattern, synchronous/mixed/alternating	3/21/7	1/4/1	2/17/6	

**TABLE 3:** Electrophysiology with tremor and dystonia involving same or different body regions.

	Tremor & dystonia in same body region	Tremor & dystonia in different body regions	p-value * indicates significance
**Head tremor**			
	n = 66	n = 3	
Frequency in Hz, mean ± SD	4.0 ± 0.9	5.0 ± 0.3	0.01*
Amplitude in g^2^/Hz, mean ± SD	0.005 ± 0.009	0.006 ± 0.006	0.11
Half peak bandwidth/irregularity in Hz, mean ± SD	0.5 ± 0.8	0.5 ± 0.6	0.75
EMG burst duration in ms, mean ± SD	101.3 ± 35.3	99.5 ± 14.2	0.054
**Arm tremor**			
	n = 31	n = 6	
Frequency in Hz, mean ± SD	5.0 ± 0.1	5.7 ± 0.8	0.06
Amplitude in g^2^/Hz, mean ± SD	0.06 ± 0.1	0.04 ± 0.1	0.051
Half peak bandwidth/irregularity in Hz, mean ± SD	0.5 ± 0.3	0.5 ± 0.4	0.81
EMG burst duration in ms, mean ± SD	132.4 ± 40.3	127.2 ± 31.8	0.21

## Data Availability

The original contributions presented in the study are included in the article/supplementary material, further inquiries can be directed to the corresponding author.
